# Manners

**Published:** 1879-04

**Authors:** 


**Manners.**—I wish cities would teach their best lesson, of quiet manners. It is the foible, especially of American youth, —pretension. The mark of the man of the world is absence of pretension. He does not make a speech; he talks in a low business tone, avoids all brag, is nobody, dresses plainly, promises not at all, performs much, speaks in monosyllables, hugs his fact. He calls his employment by the lowest name, and so takes from evil tongues their sharpest weapon. His conversation clings to the weather and the news, yet he allows himself to be surprised into thought, and the unlocking of his learning and philosophy. How the imagination is piqued by anecdotes of some great man passing incognito, as a king in gray clothes! of Napoleon affecting a plain suit at his glittering levée! of Burns, or Scott, or Beethoven, or Wellington, or Goethe, or any container of transcendent power, passing for nobody! of Epaminondas, "who never says any thing, but will listen eternally!" of Goethe, who preferred trifling subjects and common expressions in intercourse with strangers, worse rather than better clothes, and to appear a little more capricious than he was! There are advantages in the old hat and box-coat. I have heard that throughout this country, a certain respect is paid to good broadcloth; but dress makes a little restraint; men will not commit themselves.

